# International Evidence on the Impact of Health-Justice Partnerships: A Systematic Scoping Review

**DOI:** 10.3389/phrs.2021.1603976

**Published:** 2021-04-26

**Authors:** Sarah Beardon, Charlotte Woodhead, Silvie Cooper, Elizabeth Ingram, Hazel Genn, Rosalind Raine

**Affiliations:** ^1^ Department of Applied Health Research, University College London, London, United Kingdom; ^2^ Department of Psychological Medicine, King’s College London, London, United Kingdom; ^3^ Faculty of Laws, University College London, London, United Kingdom

**Keywords:** social welfare, legal services, integrated care, social determinants of health, health inequalities, health-justice partnerships, medical-legal partnerships, delivery of healthcare

## Abstract

**Background:** Health-justice partnerships (HJPs) are collaborations between healthcare and legal services which support patients with social welfare issues such as welfare benefits, debt, housing, education and employment. HJPs exist across the world in a variety of forms and with diverse objectives. This review synthesizes the international evidence on the impacts of HJPs.

**Methods:** A systematic scoping review of international literature was undertaken. A wide-ranging search was conducted across academic databases and grey literature sources, covering OECD countries from January 1995 to December 2018. Data from included publications were extracted and research quality was assessed. A narrative synthesis approach was used to analyze and present the results.

**Results:** Reported objectives of HJPs related to: prevention of health and legal problems; access to legal assistance; health improvement; resolution of legal problems; improvement of patient care; support for healthcare services; addressing inequalities; and catalyzing systemic change. There is strong evidence that HJPs: improve access to legal assistance for people at risk of social and health disadvantage; positively influence material and social circumstances through resolution of legal problems; and improve mental wellbeing. A wide range of other positive impacts were identified for individuals, services and communities; the strength of evidence for each is summarized and discussed.

**Conclusion:** HJPs are effective in tackling social welfare issues that affect the health of disadvantaged groups in society and can therefore form a key part of public health strategies to address inequalities.

## Introduction

Social welfare is a diverse area of civil law that includes issues such as welfare benefits, debt, housing, education and employment, among others. Social welfare legal problems are known to be harmful to health: population surveys of legal need have shown direct impacts such a stress-related illnesses and physical ill health [1, 2]. Indirect effects can occur through the consequences of legal need such as poverty, homelessness, poor living and working conditions. In the public health discourse, these circumstances are understood as “social determinants of health,” which are major causes of illness and inequality internationally [3]. Indeed, the World Health Organization estimates that income security and living conditions account for almost two thirds of health inequities between socioeconomic groups within countries of the European region [4]. Optimizing people’s access to the protections afforded them under social welfare law is therefore highly relevant to public health as a means of preventing disease, improving health and reducing health inequities. This can be facilitated by services offering advice and assistance on social welfare legal rights.

Partnerships between healthcare and legal services have emerged across the world in response to the close relationship between health and social welfare issues [5–7]. A wide range of service models exists, including co-located services, referral pathways and integrated multidisciplinary teams [8]. For the purposes of this review, ‘health-justice partnership’ is defined broadly as the provision of legal assistance for social welfare issues in healthcare settings.

Health-justice partnerships (HJPs) have potential to generate outcomes that are important policy objectives for both health and legal sectors. In the health field, forming integrated service partnerships is promoted as a means to address social determinants of health and improve the wellbeing of populations [9]. In the legal field, integrating free legal assistance within other community-based services is promoted as a means to facilitate timely access to appropriate legal help [10]. HJPs also have potential benefits for both health and legal practitioners. Patients frequently present to healthcare professionals with social welfare problems, which may result from their health condition or be contributing to their illness [11]. Partnerships with legal services can assist healthcare professionals to address the social welfare needs of patients, which are beyond their expertize to manage [12]. For legal practitioners, partnerships with healthcare could facilitate intervention at an earlier stage before social welfare problems escalate [13] and can enable access to the medical information needed to support welfare casework and to advocate for systemic change [14, 15]. On an individual level, patients stand to benefit from a coordinated response to their needs, with support for both health and welfare issues [16].

Understanding the impacts of HJPs is important given the many potential benefits of these service models. International evidence on the impacts of HJPs has not been systematically reviewed. Previous reviews have focused on specific regions and service models [17, 18], or have not applied systematic methods [19, 20]. A systematic scoping review was undertaken to map international evidence on the delivery of HJPs across a range of topics. This paper focusses on service impacts, answering the following research questions:

i.What are the key objectives of HJPs?
ii.What is the range and strength of evidence to demonstrate outcomes against each of these key objectives?

## Methods

### Methodological Approach

Scoping reviews involve undertaking broad assessments of available evidence in areas where the literature has not previously been characterized [21]. The method used for this scoping review followed the steps outlined in the guidance published by Arksey and O’Malley 2005 [22] and Levac, Colquhoun and O’Brien 2010 [23].

### Search Strategy

All literature sources are detailed in Supplementary Appendix 1. Literature was sought for the dates January 1995–December 2018, covering the period since HJP services were first reported. Twelve academic databases were examined, encompassing the fields of medicine, law, health management and social science. Grey literature was also sought: websites of relevant organisations were searched, including health-justice organisations, legal charities, legal services’ networks and public bodies in health, social care and law. Reference lists of included studies were scanned to identify additional citations.

The following key concepts were used to develop search terms: “social welfare legal advice” AND “healthcare” OR “health-justice partnership”. For full search terms see Supplementary Appendix 1. Keyword search term combinations reflecting these concepts were developed in Ovid Medline and applied across all databases. Indexing terms were also applied in each database, including “Civil rights,” “Legal services,” “Social welfare,” “Health services” and “Delivery of healthcare.” The same terms were used to search websites for gray literature.

### Study Selection

Records retrieved from the academic databases were exported to Endnote software and duplicates removed. Records were selected based on the relevance of the title, followed by the abstract and full text. Inclusion and exclusion criteria are specified in Table 1. Reasons for exclusion were recorded during full text assessment. The selection process was repeated by a second reviewer with a random 10% sample of the full texts obtained. Any disagreements were resolved through discussion.

**TABLE 1:** Study selection criteria.

	Inclusion	Exclusion
Service definition	Services providing legal assistance with social welfare issues in healthcare settings (direct physical or functional link between legal and healthcare service)	Areas of law other than social welfare
		Information or advocacy services (not legal assistance)
		No direct links with healthcare
Language	Publications printed in English	
Publication date	Publication date between 1st January 1995 and 13th December 2018	
Geographical location	OECD countries	
Research type	Primary studies of any research design (both quantitative and qualitative), reviews and grey literature reports	Publications not presenting empirical findings, publications presenting vignettes only
Publication type	Peer reviewed journal articles, reports, service evaluations	Editorials, discussion papers, opinion pieces, letters and commentaries, conference abstracts

### Data Extraction and Quality Assessment

Key information relevant to the review questions was extracted from the publications and entered into a spreadsheet for analysis. This included publication characteristics, details of service design and delivery, study research methods and reported results. A quality assessment tool was developed using items drawn from existing checklists (Supplementary Appendix 2). Existing tools could not appropriately be applied given the unique combination of disciplines and the diversity of research designs and literature types included.

### Analysis

A narrative synthesis was used to characterize the literature and summarize key findings, integrating both qualitative and quantitative data [24]. Findings were synthesized in relation to the review questions: i) information on the objectives of HJPs was categorized thematically and the frequency of each theme reported in the literature was counted; ii) data on the measured outcomes were summarized narratively against each objective and quality of the evidence discussed.

## Results

**FIGURE 1:**
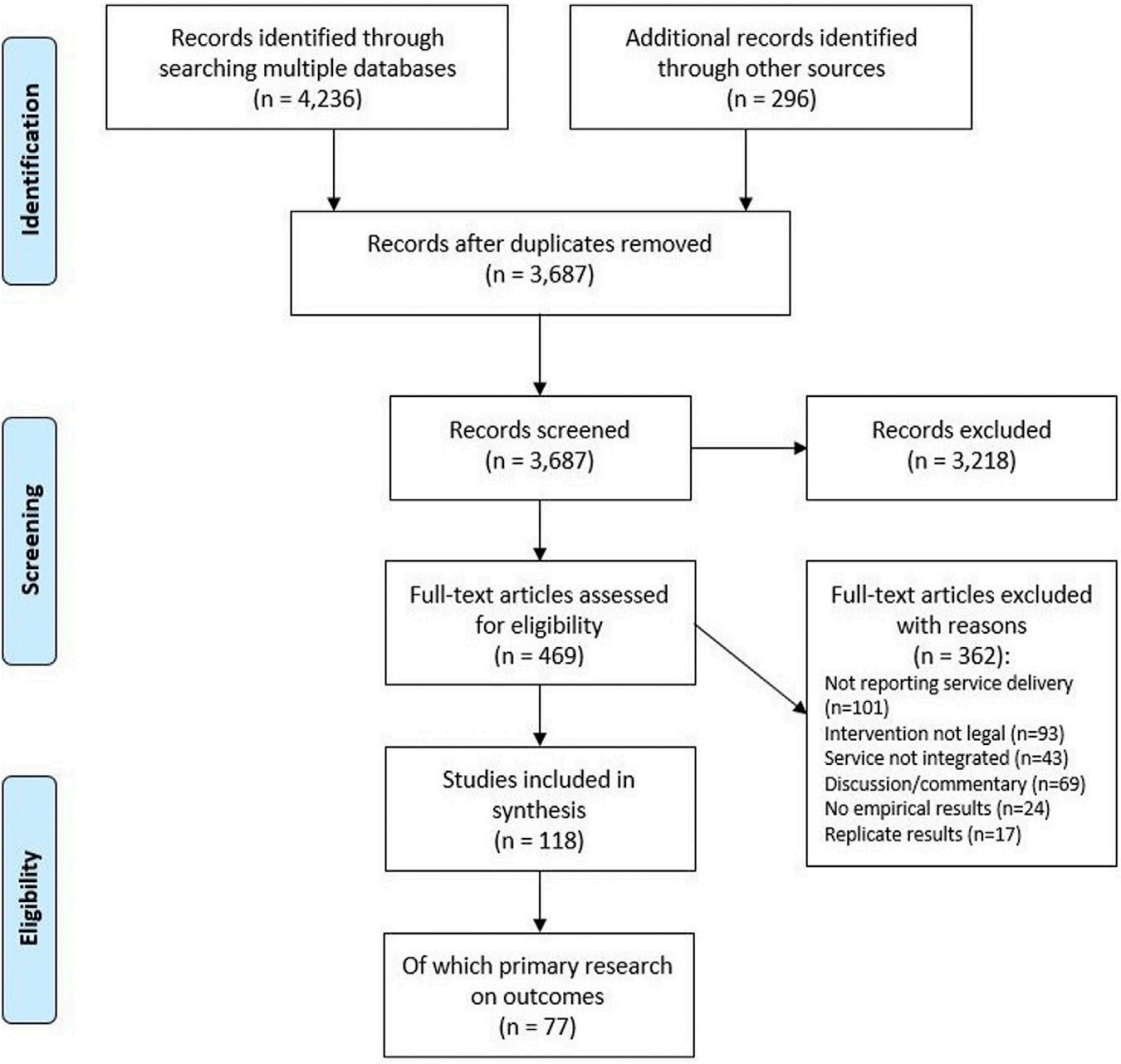
Search and screening process.

Searches of academic databases, gray literature and other sources returned 3,687 records, of which the full text of 469 articles were screened against inclusion and exclusion criteria. The selection process led to a final sample of 118 publications included (Figure 1).

### Publication Characteristics

Table 2 presents characteristics of the included publications. They originated predominantly from the United Kingdom (n = 60) and United States (n = 43). The majority reported primary research studies (n = 87) and were published in peer-reviewed journals (n = 69).

**TABLE 2:** Characteristics of included publications.

		Count (total N = 118)	%
Country of origin	United Kingdom	60	51
	United States	43	36
	Australia	9	8
	Canada	4	3
	Ukraine	1	1
	New Zealand	1	1
Study type	Primary research	87	74
	Descriptive report	23	19
	Evidence review	4	3
	Other	4	3
Publication type	Peer-reviewed journal article	69	58
	Organisational report	42	36
	Other grey literature	7	6

### Service Characteristics

Reports that mapped characteristics of HJPs in various countries demonstrated their broad diversity [6, 8, 25]. Target populations commonly focused on low income or disadvantaged groups, people with specific health conditions (e.g. cancer, mental health) or demographic characteristics (e.g. children, the elderly, the homeless). Healthcare settings included primary, secondary and specialist care. Legal assistance was provided free for clients, largely by charitable and non-profit organisations, and could be either specialist or generalist in nature. Social security and other financial issues were the most commonly reported focus but a wide range of other social welfare issues were addressed including housing, employment and family stability.

Approaches to linking delivery of healthcare and legal services varied: co-location (being physically located together) and referral pathways were common in all geographical regions; other approaches included incorporating legal advisors into multi-disciplinary teams [26, 27], integrating legal support into care pathways [28], and undertaking joint clinics or assessments [29, 30]. Technology-based approaches also existed, such as providing patients with direct-access telephones to welfare advisors [31]. Screening for health-harming legal needs was commonly practiced in the United States but was not widely reported in other regions [32].

### Partnership Objectives

Objectives of HJPs reported in the literature fell into a number of broad themes (Table 3). These were: prevention of health and legal problems; access to legal assistance; health improvement; resolution of legal problems; improvement of patient care; support for healthcare services; addressing social inequalities; and catalyzing systemic change.

**TABLE 3:** Objectives of health-justice partnerships.

#	Theme	Description of objectives	Count (N)
1	Prevention of health and legal problems	To address underlying causes of ill health (health-harming socioeconomic and environmental factors), prevent ill health and deterioration, provide early legal intervention and prevent crisis situations developing	37
2	Access to legal assistance	To facilitate access to legal assistance, reach those in greatest need and those who may otherwise have difficulty obtaining legal help	34
3	Health improvement	To improve health (both physical and mental), improve wellbeing and quality of life, support recovery, alleviate stress and its impact on health	30
4	Resolution of legal problems	To address legal problems, alleviate poverty and social disadvantage, help individuals attain their rights and improve uptake of welfare entitlements	29
5	Improvement of patient care	To provide a high standard of support, improve integration and fill gaps in care, respond holistically to inter-connected issues through collaborative working, increase knowledge and capacity of services by combining expertize of professions	28
6	Support for healthcare services	To address non-medical needs of patients, provide a resource for healthcare professionals, free up clinical time, improve efficiency and reduce demand on healthcare	18
7	Address inequalities	To reduce social and health inequalities and increase social inclusion by addressing underlying disparities in socioeconomic conditions	15
8	Catalyze systemic change	To use legal advocacy to address systemic issues affecting the health of populations	7

### Partnership Outcomes

77 publications reported results of primary research assessing outcomes of HJPs; these are considered in the following narrative synthesis. Broad characteristics of the 77 studies are presented in Table 4 and details of each paper are presented in Supplementary Appendix 3. Findings are presented according to the service objectives.

**TABLE 4:** Characteristics of primary studies reporting service outcomes.

Characteristic		Count (N)
Healthcare setting	Primary care	36
	Hospital care	18
	Community care	12
	Multiple	11
Study type	Observational	73
	Experimental	2
	Quasi-experimental	1
	Other	1
Data type	Mixed methods	36
	Quantitative	31
	Qualitative	10
Research design	Retrospective record review	36
	Cross-sectional study	24
	Pre-post follow-up	13
	Modeling	2
	Comparative case study	1
	Unspecified	1
Quality rating^a^	Low	7
	Low/Medium	25
	Medium/High	33
	High	12

#### Prevention of Health and Legal Problems

Several high quality qualitative studies conducted in the United Kingdom primary care context have found that additional income gained as a result of welfare rights interventions was commonly spent on settling bills such as fuel payments, and affording more or better quality food [16, 33–37]. The extra income enabled people to get out more, participate in daily activities and maintain social contact by covering the costs of transport and social activities [33–37] and for some, it enabled access to paid-for health services such as dentistry, eye care and home help [16, 33, 36]. Successful welfare claims were a gateway to other forms of non-financial help, such as free prescriptions, respite care, meals on wheels and home modifications [38]. Reduced financial pressure had benefits for personal independence and eased strain on family relationships [37, 38]. High quality quantitative surveys of clients accessing welfare rights advice in United Kingdom primary care settings found self-reported improvements in knowledge, empowerment and confidence as a result of the interventions [39, 40]. Qualitative evidence reflects this: interview studies have identified increased confidence and empowerment resulting from welfare rights interventions [14, 41–43], leading to improved ability to use other services [14], coming off drugs and entering education and training [43], being more open with healthcare staff about their situations [42], and being able to focus on their health [41]. A small-scale survey conducted in the United States showed significant reductions in the proportion of families avoiding healthcare for their children due to financial concerns [44].

#### Access to Legal Assistance

High-quality studies conducted in United Kingdom primary care found that people referred to advice by healthcare professionals would not otherwise have sought assistance [31, 38, 45]. Qualitative evidence showed that healthcare-based provision facilitated access for certain groups such as older people [46–48] and those in poor mental and physical health [14, 45, 49]. Studies of service user experiences identified that the healthcare environment was conducive to seeking help with legal issues because it felt familiar and trusted, discreet and confidential, less stigmatized, often less far to travel and somewhere people felt comfortable discussing anxieties [16, 31, 45, 48, 49]). Referrals from primary care staff encouraged help-seeking, legitimizing the receipt of welfare assistance as part of a wider holistic approach to care [31, 33, 38]. The trusting relationship with healthcare professionals facilitated patients’ engagement with legal advisers [14, 48, 50]. Studies conducted in United Kingdom cancer services highlighted that patients with serious illness may not have the physical or mental strength to pursue legal processes or may assume they are not entitled to help unless alerted by healthcare professionals [51, 52]. Quantitative outcomes reflect similar themes: surveys of clients accessing welfare support in United Kingdom primary care estimated that 66% would not have accessed assistance had they not been referred by a healthcare professional [53], and that almost half (45–49%) of HJP clients would be unlikely to seek advice elsewhere [39, 54]. Features of the healthcare setting that clients rated ‘very important’ to them included closeness (78%), a place they trusted (80%), a place they knew (73%), and that it was anonymous (43%) [39]. In a United States pediatric hospital setting, 85% of clients had not used legal resources before accessing the service, and 79% had not been aware of legal resources [44].

#### Health Improvement

Experimental studies of health outcomes had only been conducted in the United Kingdom primary care setting: two papers reported pilot randomized controlled trials, of which one was insufficiently powered for statistical analysis [29]. The other found little evidence of any changes over time (at 24 months following the intervention) or differences between intervention and control groups across a range of health, behavioral and psycho-social outcomes; however, study design limitations may have affected the potential to demonstrate change [55]. A quasi-experimental study explored the effects of co-located welfare rights advice in primary care compared with a propensity score-weighted comparison group [40]. This study showed an improvement in mental wellbeing among individuals whose situation improved as a result of advice, significant reductions in rates of common mental disorders among women and participants of a Black/Black British ethnicity, and improvements in stress levels.

Uncontrolled prospective studies have been conducted in a variety of settings. In United Kingdom primary care, improvements in mental health and emotional role functioning were found where income had increased as a result of financial interventions [33, 34]. A small-scale study conducted in a hospital setting in the United States showed significant reductions in asthma severity and medication usage for adult asthma patients receiving a housing intervention [56]. In a family medicine clinic in the United States, perceived stress among adult patients or carers reduced significantly following receipt of legal assistance, and this change was strongly associated with the level of concern regarding legal issues [57]. In veterans’ medical centers in the United States, veterans receiving a greater level of input from legal services showed greater improvements in mental health and general health scores [58].

Qualitative studies conducted with patients receiving legal assistance in a variety of healthcare settings internationally have reported reduced feelings of stress and anxiety [16, 36–38, 42, 51], improved mental stability [16, 38], greater peace of mind and reassurance [35, 36, 51], hope [42], better sleeping [16, 38], improved wellbeing and quality of life [36, 37] and increased ability to cope with ill health [36, 37]. Two papers developed theories of change as to how welfare advice interventions may lead to improved health [14, 52]: the models propose that legal assistance brings about improved circumstances (material, financial and practical) which leads to reduced stress and anxiety, improved ability to focus on health and participate in daily life, and ultimately better mental and physical wellbeing. There were fewer indications in the qualitative studies of perceived impacts on physical health. One good quality paper from a United Kingdom primary care setting described patients reporting heathier behaviors, including reduction or cessation of smoking, improved diet and physical activity, reversal of weight loss and changes in medication [38], but no other studies have confirmed these findings.

#### Resolution of Legal Problems

Two studies have assessed legal outcomes against a comparison group, both focusing on welfare rights interventions in the United Kingdom primary care setting; they found significantly greater improvements in financial strain [40] and financial vulnerability [55] in the following months for people receiving the intervention. Studies conducted in a variety of settings have highlighted high success rates for legal assistance in obtaining welfare support and increasing the incomes of recipients [33, 34, 36, 52, 59–61]. Internationally, reports consistently showed significant amounts of money were received as a result of legal assistance, as lump sums and regular ongoing contributions to income [31, 35, 36, 47, 50, 52, 59–68]. Other financial outcomes included preventing benefits stoppage [59], managing debts [50, 65], reducing use of credit cards [40] and obtaining access to healthcare insurance [69, 70]. Qualitative research has highlighted the importance of this financial assistance in easing difficult situations and helping to mitigate the financial consequences of illness [38, 52]. Other legal issues resolved successfully through HJP interventions internationally included housing circumstances and homelessness [40, 63, 69, 71, 72], education [63, 69, 71, 72], family stability [63, 69], employment [63], wills and power of attorney [66], utility shut-offs [73] and food supports [44, 63].

#### Improvement of Patient Care

Feedback gathered from project staff in a variety of international settings suggests that HJPs provide a more rounded service for patients, addressing interconnected health and welfare issues in a comprehensive way [5, 16, 74]. Partnership working between health and legal services helped to resolve issues affecting health and wellbeing and was felt to make a positive contribution to patient care [45, 75]. Patients reported valuing the continuity of support, familiarity and personalized service [16]. Those with serious illness felt that proactive assistance with social welfare rights issues was an important part of non-medical care, and should be made available to support patients [51]. Studies reporting views of clinicians have highlighted that being able to offer legal support can improve patients’ confidence and trust in the health service and contribute to stronger doctor-patient relationships [50, 54].

#### Support for Healthcare Services

Studies had investigated whether HJPs could reduce pressure on health services by reducing care utilization. The only experimental study investigating this outcome did not have sufficient statistical power to show significant changes [29]. A quasi-experimental controlled study found no significant changes in primary care consultation rate in response to a welfare rights intervention in the United Kingdom [40]. Evidence from uncontrolled follow-up studies did not show a consistent pattern: two studies suggested reductions in service use, in response to a housing intervention for asthma patients delivered in a hospital setting in the United States [56] and welfare rights advice delivered in a United Kingdom primary care context [76]. However, others have found no significant changes, including in response to welfare rights advice in United Kingdom primary care [33] and legal assistance for low income families in a children’s hospital in the United States [44]. One study identified instances of earlier discharge from a United Kingdom hospital: financial awards had enabled patients to secure suitable accommodation and necessary care packages to return home from intensive care [77].

In the United States where access to health insurance is not universal, legal services had obtained insurance cover for patients and intervened against complex insurance denials, thereby facilitating access to needed healthcare [69, 70]. HJPs focusing on patient access to health insurance in the United States have been found to generate significant sums of money for hospitals through health insurance reimbursements [78]. This supports return on investment by the healthcare partner and allows patients to engage with preventative health care, reducing the likelihood of future health emergencies [78]. A qualitative study from the Ukraine found that providing legal assistance in harm reduction services for drug users led to increased engagement with preventative healthcare among this group [41].

High quality studies exploring perspectives of healthcare professionals in the United Kingdom and Canada report that partnerships with legal services can be a beneficial resource to support them in their work: clinicians reported that these partnerships provide an opportunity to address patients’ non-medical issues outside their expertize [14, 16, 33, 49] and that this was potentially time-saving as it meant they did not have to address legal issues themselves and could focus on individuals’ health and care needs [14, 16, 42, 49]. Healthcare professionals have been found to report better job satisfaction as a result of partnerships with legal services, due to feeling able to perform their role more effectively [14] and feeling satisfied at providing a good service for patients [33, 42, 49].

#### Addressing Inequalities

Studies had not specifically investigated whether HJPs were effective in reducing health or social inequalities. One study investigated differential mental health outcomes across gender, ethnicity and health status; it found that women and participants of a Black/Black British ethnicity were particularly likely to benefit in terms of common mental disorders as a result of a welfare rights intervention in United Kingdom primary care [40]. Targeting housebound patients resulted in greater financial benefit for this group than for patients attending surgery-based welfare rights advice sessions [31].

#### Catalyzing Systemic Change

HJPs occupy a unique position at the intersection of health and rights [79], which enables them to identify patterns of discriminatory or harmful practices and community-level health risks [41, 80, 81]. Case studies from the United States demonstrate a number of ways in which partnerships have addressed population-level health risks, including through action against landlords to improve living conditions [71], changes in legislation that include new health and safety laws [82], provision of adequate services for people with disabilities and mental illness [83], and extra protection for vulnerable groups facing utility shut-offs [84]. Contributing to court cases, government enquiries and public consultations is another way that partnerships have exerted influence at systemic level. Examples from countries across the world highlight the impact of these activities in contributing to changes in the welfare eligibility laws [15], giving voice to vulnerable groups in the legislation process [43], informing organisational responses to family violence and elder abuse [85] and contributing to human rights work for families and children [86].

## Discussion

### Main Findings

This systematic scoping review identified the stated objectives of Health Justice Partnerships (HJPs), and mapped the international evidence on impact against each key theme. There was strong evidence for their effectiveness in resolving legal problems and thereby improving the socioeconomic circumstances of individuals, outcomes that were reported from all regions and service types. This demonstrates the important role of HJPs in addressing social determinants of health, a cornerstone of public health policy in health systems across the world [87]. There was also strong evidence that HJPs improve access to legal assistance for patient groups that would otherwise not seek help for social welfare issues. HJPs therefore facilitate action on health and social inequalities by reaching those most likely to be affected by health-harming legal need [88].

The impacts of HJPs on individual health has been the subject of debate [89]*.* The reviewed publications had examined different health outcomes (mostly self-reported), among different patient groups, for different legal interventions and over different time periods. Broad generalization is therefore not possible from the current evidence. Health impacts are likely to depend on the patient population (e.g. age, health status) and legal issues addressed. However, overall there was strong evidence among the studies (both quantitative and qualitative) for improvements in mental health, particularly stress, depression, anxiety and wellbeing, and that these improvements occurred as a direct result of the legal interventions [16, 35–38, 40]. Of the literature reviewed in this study, only three papers used a control or comparison group to assess changes in health; these were all high quality peer-reviewed publications from the United Kingdom undertaken in a primary healthcare setting [29, 40, 55]. Since the literature search was conducted, two further studies reporting results of randomized controlled trials have become available. Howel et al. 2019 [90] found no effect on a range of health outcomes among people aged ≥60 years receiving welfare rights assistance delivered through primary care in the United Kingdom; however, a true effect may have been masked by poor intervention targeting and contamination between trial arms. Bovell-Ammon et al. 2020 [91] found significant improvements in parent and child health among medically complex families receiving a housing stability intervention via healthcare settings in the United States. This was a multi-component intervention and the study was relatively small, therefore the effects of the legal assistance could not be separated out; however the overall findings showed significant improvements in both mental and physical health compared with families not receiving the housing intervention.

There were some areas where the evidence was of lower quantity and quality. For example, no studies had assessed prevention directly, although many provided evidence of wider social benefits which could prevent ill health in the long term (such as improved living conditions, social participation and access to supportive services). Few studies had measured direct effects on inequalities; however, the benefits of HJPs as a whole are likely to accrue to those of lower socioeconomic status given the nature of the social welfare issues they address and their focus on low income and disadvantaged groups. Studies reporting impacts on health service utilization showed inconsistent patterns and mostly lacked appropriate comparative evidence. This outcome is likely to depend on the characteristics of local services (e.g. target patient groups, legal issues addressed, type of service model) and further research would be needed to investigate how health service utilization outcomes may be influenced by the service context. The opposite goal (increased health service use) is relevant in situations where patients may face legal or social barriers to access, and the studies highlighted a role for HJPs in facilitating engagement with needed healthcare. Other impacts for health services and patient care had been explored to a lesser extent and were not the focus of much high quality research; benefits identified qualitatively included supporting healthcare professionals to manage patients’ non-medical needs and improving both practitioner and patient experience. Catalyzing systemic change through legal and policy action was more rarely reported in the literature, however case studies demonstrated the wide-reaching effects of these activities in protecting the health of populations.

### Strengths and Limitations

This paper presents the first systematically conducted review to include publications from across international regions and to consider a range of service models and settings. The review drew on a wide-ranging systematic search that included both academic and grey literature, ensuring evidence from practice was included alongside academic research. This broad scope means that service objectives and impacts are reported comprehensively and the full range of diverse HJPs are represented. The study selection process was verified by a second reviewer to ensure the inclusion and exclusion criteria were applied accurately. However, it is unlikely that every paper on the topic was uncovered, particularly in the grey literature where relevant international sources may have been unknown to the authors. The quality assessment checklist was developed by the lead author and has not been validated formally as a tool. It enabled a consistent approach for quality assessment across all the papers, but offers a general rather than specific estimate of quality given the range of disciplines, study types and outcomes it was designed for.

### Practice Implications

This review demonstrates the potential of HJPs in addressing interconnected health and welfare issues at the level of individuals, services and communities. With the current Covid-19 pandemic following a decade of global austerity, social welfare legal need in the population is likely to rise. Worsening economic and social conditions may lead to and exacerbate long term health consequences (especially for mental health) and widening inequalities [92]. As with the effects of previous recessions, social welfare-related workload could increase for healthcare professionals, placing additional strain on health services [93, 94]. HJPs offer a means to assist healthcare professionals in addressing social welfare legal needs among patients, providing more responsive care and better supporting individuals whose health is affected by adverse socioeconomic circumstances. HJPs therefore facilitate action both on health inequalities and access to justice [88].

## Conclusion

This review summarizes the objectives of HJPs and assesses the strength of international research evidence on service impacts. There is strong evidence that HJPs are effective in reaching people at risk of social and health disadvantage, positively influencing social determinants of health through the resolution of legal problems, and improving mental wellbeing. A wide range of other benefits for individuals, services and communities are identified and discussed. HJPs have an important role to play in tackling the social determinants of health and should be considered in public health strategies addressing health and social inequalities.

The review also highlights areas that future research could build on. Use of robust study designs with comparator groups would strengthen current evidence of effectiveness. Randomized controlled trials have been hampered by design issues when used to investigate these complex interventions, but alternatives such as natural experiments and use of routine data sources offer an alternative approach for robust evaluation. Outcomes such as health improvement and healthcare utilization are likely to depend on population groups and local service models; comparative studies would help identify how outcomes may vary by context. Impacts that could benefit from further investigation include the role of HJPs in prevention and early access to services (both health and legal), their contribution to patient care (such as engagement and longer-term trajectories), health inequalities (differential outcomes across social groups) and their role in health service functioning (for example, efficiency, effectiveness and patient experience).
