# Supplementary material for: International Evidence on the Impact of Health-Justice Partnerships: A Systematic Scoping Review
Source: Public Health Rev. 2021 Apr 26;42:1603976. doi: 10.3389/phrs.2021.1603976 (PMC8113986; doi:10.3389/phrs.2021.1603976)
Supplement: Supplementary file 1 [file DataSheet2.PDF]

APPENDIX 2: QUALITY ASSESSMENT

Methods for development of quality assessment tool:

The quality assessment tool was developed for the body of literature gathered in this review, reporting on the delivery of HJPs. Existing tools could not easily or appropriately be applied, given the unique combination of disciplines and the diversity of research designs and literature types included. Quality assessment frameworks were reviewed to identify ratings of reporting that could be applied across the papers as a whole; these included frameworks from the medical, legal and interdisciplinary fields(Centre for Evidence Based Medicine, no date; Critical Appraisal Skills Programme, no date; UK Government, 2014; Jenkins, Partin and Wise, 2015; Belcher *et al.*, 2016; Mårtensson *et al.*, 2016; Van Gestel, Byland and Lienhard, 2018). Items from these checklists were first listed in full, and any that were too specific to a method or discipline were excluded. Remaining items were grouped according to similarity of meaning, and assessed as to how appropriately they could be applied to the included papers. This process of reduction resulted in a series of 16 statements relating to the following aspects of the papers: 'Context', 'Methods', 'Reporting', and 'Formal assessments'. Each criterion was rated using the following scores: 0 = 'No evidence', 1 = 'Some evidence', 2 = 'Good evidence'. Scores were then summed to give an overall value on the scale of 0 – 34. This was converted to a broader rating category: 0-10=Low, 11-20=Low/Medium, 21-30=Medium/High, 30+=High.

Table 1: Quality assessment tool developed

	**Criteria**	**Meaning**
**Context**	Engagement with existing evidence and theory	Adequately presents existing knowledge/ research evidence relevant to the context<br>Demonstrates understanding of the relevant issues and sources<br>Sets out hypotheses / theories of change
	Clear description of context/setting	A detailed description of the setting and context in which the service operates<br>e.g. details of location, health service, legal service, target population, service aims
	Clear description of intervention	A detailed description of the activities of the service<br>e.g. legal intervention, links with healthcare, operational details
**Methods**	Relevant research problem	Research problem is clearly stated and defined.<br>Research problem is grounded in the context (including current debates, social context, knowledge gaps, theories).<br>Practical applications of the research are considered.
	Clear definition of research aims/questions	Research aims and/or questions are clearly stated and defined.<br>Research questions are related to the problem context.
	Appropriate study design/methodology	The processes of undertaking the research are clearly described.<br>The study design is appropriate for addressing the research questions.<br>The methods fit the purpose and are sufficient to generate the evidence required.

	Appropriate selection of data subjects/ sources	Selection of data subjects and/or sources is well described<br>Sampling procedure is inclusive of all relevant participants / data sources
	Appropriate information gathering	Data collection procedures are clearly described<br>Choice of outcome measures is fitting and sufficient<br>Data collection is of sufficient duration and completeness
	Rigorous analysis	Analysis of study data is clearly and comprehensively explained<br>Appropriate analysis methods are employed
	Use of theoretical foundation	The approach is informed by appropriate theories relevant to the context<br>Theories are developed or examined
**Reporting**	Clear and comprehensive reporting	Results are clearly presented in full<br>Language is clear, precise and understandable<br>Report is well structured
	Critical evaluation	Information is critically evaluated<br>Potential bias or confounding is discussed and taken account of if possible<br>Alternative explanations are considered
	Critical reflection	Limitations of the research are discussed<br>The role/standpoint of the researcher is disclosed and its impact considered<br>Potential influence of context-specific cultural factors is considered
	Valid conclusions	Conclusions are linked to study's results and other existing evidence<br>Interpretation is logical and transparent<br>Argument is well-crafted
	Transferability	The research makes a significant contribution to knowledge or practice<br>The application of the findings in other contexts is discussed (generalisability)
**Formal assessments**	Peer reviewed	The publication has undergone peer review
	Consideration of ethical issues	Ethical challenges are considered and responded to<br>The research has undergone a process of ethical review
