# Supplementary material for: International Evidence on the Impact of Health-Justice Partnerships: A Systematic Scoping Review
Source: Public Health Rev. 2021 Apr 26;42:1603976. doi: 10.3389/phrs.2021.1603976 (PMC8113986; doi:10.3389/phrs.2021.1603976)
Supplement: Supplementary file 3 [file DataSheet3.PDF]

APPENDIX 3

Table 1: Characteristics of studies reporting HJP service outcomes

**Publication**	**Service setting**	**Study design**	**Outcomes reported (themes)**	**Peer reviewed?**	**Quality rating**
Abbott 1999	UK, primary care, welfare benefits advice delivered in health centres	Observational, Mixed methods. Retrospective review of service records, before-and-after follow-up surveys with clients, interviews with service users and primary care staff	Access to assistance, Legal, Health, Prevention, Healthcare support	No	Medium/High
Abbott et al. 2006	UK, primary care, welfare benefits advice delivered in general practices	Observational, Quantitative. Before-and-after follow-up, structured interviews with clients	Legal, Health, Prevention	Yes	Medium/High
Abbott and Hobby 2003	UK, primary care, welfare benefits advice delivered in general practices	Observational, Quantitative. Cross-sectional survey of clients	Access to assistance, Legal, Health	Yes	Medium/High

Atkins et al. 2013	USA, women's healthcare, legal services for pregnant women on low incomes	Observational, Quantitative. Before-and-after follow-up survey with clients	Health	No	Low/Medium
Bateman 2008	UK, variety of healthcare types, welfare benefits advice delivered in healthcare settings	Observational, Mixed methods. Cross-sectional survey of advice providers, interviews with project staff, healthcare professionals and service users	Access to assistance, Legal, Health, Care delivery	No	Low/Medium
Beck et al. 2012	USA, children's hospital, legal services to address housing problems for low income families in poor living conditions	Observational, Quantitative. Retrospective review of service records	Legal, Systemic change	Yes	Medium/High
Bird 1998	UK, mental health services, welfare advice and assistance delivered in health settings	Observational, Qualitative. Cross-sectional, interviews with clients, advice staff and mental health professionals	Access to assistance	Yes	Low/Medium

Burrows et al. 2011	UK, primary care, welfare rights advice in general practices	Observational, Qualitative. Cross-sectional data collection, semi-structured interviews with clients, healthcare professionals and advisors	Access to assistance, Legal, Health, Prevention, Healthcare support	Yes	Medium/High
Carey and Tolopilo 2008	Ukraine, harm reduction programs and HIV/AIDS services, legal assistance for people suffering from drug dependency, people living with HIV/AIDS, and commercial sex workers	Observational, Qualitative. Cross-sectional, interviews with legal professionals, clients and service providers	Access to assistance, Prevention, Healthcare support, Systemic change	No	Low/Medium
Carrick et al. 2017	UK, primary care, welfare advice delivered in general practices	Observational, Mixed methods. Social return on investment modelling, based on service records, stakeholder consultations, client surveys and staff interviews	Access to assistance, Legal, Health, Prevention, Healthcare support	No	Medium/High
Citizens Advice and the Royal College of General Practitioners 2018	UK, primary care, welfare advice outreach sessions in GP surgeries	Observational, Mixed methods. Cross-sectional data collection, general practitioner survey, service case studies	Access to assistance, Healthcare support	No	Low

Citizens Advice Camden 2017	UK, primary care, welfare advice delivered in general practices	Observational, Mixed methods. Cross-sectional, data collection, post-intervention client survey, telephone interviews with clients and healthcare providers	Access to assistance, Legal, Health, Prevention, Healthcare support	No	Low/Medium
Community Sense 2015	UK, primary care, welfare advice outreach sessions in GP surgeries	Observational, Mixed methods. Retrospective review of service records, cross-sectional patient and staff surveys, stakeholder interviews, patient case descriptions	Access to assistance, Legal, Health, Healthcare support	No	Low/Medium
Coppel et al. 1999	UK, primary care, welfare advice delivered in a general practice	Observational, Mixed methods. Retrospective review of service records, post-intervention survey of clients, semi-structured interviews with primary care staff	Access to assistance, Legal, Care delivery	Yes	Low/Medium
Eynon et al. 2010	UK, intensive care, legal assistance relating to personal injury for patients in intensive care	Observational, Quantitative. Retrospective review of service records	Legal	Yes	Low/Medium
Fleishman et al. 2006	USA, hospital-based cancer centers, legal services for poor and chronically ill cancer patients	Observational, Mixed methods. Cross-sectional client survey	Access to assistance, Legal, Health, Care delivery	Yes	Low/Medium

Focus Consultants 2017	Canada, children's hospital, legal advice and representation for low income families	Observational, Mixed methods. Retrospective review of service records, interviews and surveys with clinicians, lawyers and clients	Access to assistance, Legal, Health, Systemic change	No	Low/Medium
Frost-Gaskin et al. 2003	UK, community mental health services, welfare benefits assistance provided in health centres for people with mental health problems	Observational, Mixed methods. Before-and-after follow-up, case histories	Access to assistance, Legal, Health	Yes	Medium/High
Gabbay et al. 2017	UK, primary care mental health services, debt advice delivered in general practices for adults with depression	Experimental, Mixed methods. Before-and-after follow up with control group, qualitative interviews with clients, clinicians and advisors	Legal, Health	Yes	High
Galvin et al. 2000	UK, primary care, welfare rights advice in general practices for patients and carers	Observational, Mixed methods. Cross-sectional data collection, client survey, interviews with clients and advisors, focus group with general practitioners	Access to assistance, Prevention, Care delivery	Yes	Medium/High

Greasley and Small 2005a	UK, primary care, welfare advice sessions delivered in general practices	Observational, Mixed methods. Cross-sectional, practice manager survey, focus groups with advisors and primary care staff	Access to assistance, Health, Prevention, Healthcare support, Care delivery	Yes	Medium/High
Greasley and Small, 2005b	UK, primary care, welfare advice delivered in general practices	Observational, Quantitative. Retrospective review of service records	Access to assistance, Legal	Yes	Low/Medium
Harding et al. 2002	UK, primary care, welfare advice sessions delivered in general practices	Observational, Quantitative. Cross-sectional survey of practice managers	Access to assistance	Yes	Medium/High
Harding et al. 2003	UK, primary care, welfare rights advice delivered in general practices	Observational, Quantitative. Cross-sectional survey of general practitioners	Access to assistance, Healthcare support	Yes	High
Hernández 2016	USA, community health centers, legal assistance for housing problems for patients on low incomes	Observational, Qualitative. Comparative case study, semi-tructured interviews with clients and comparison group	Access to assistance, Legal	Yes	Low/Medium

Hoskins et al. 2005	UK, primary care, welfare benefits screening and advice for patients aged over 64 years with care needs	Observational, Quantitative. Prospective collection of service process and outcome data	Access to assistance, Legal	Yes	Medium/High
Hoskins and Smith 2002	UK, primary care, welfare rights assessment and assistance for physically or mentally frail patients aged 65 years and over	Observational, Mixed methods. Retrospective review of service records	Access to assistance, Legal	Yes	Medium/High
Jackson et al. 2012	Canada, children's hospital, legal assistance for low income families	Observational, Mixed methods. Retrospective review of service records, semi-structured interviews with healthcare providers, clients and lawyers	Health, Prevention, Healthcare support	No	Medium/High
Jones et al. 2017	Canada, primary care, income security intervention offering expert advice, advocacy and case management	Observational, Quantitative. Retrospective review of service records	Access to assistance, Legal	Yes	High

Justice Connect Seniors Law and cohealth 2016	Australia, community health service, legal assistance for older people experiencing elder abuse and other legal issues associated with ageing	Observational, Mixed methods. Before-and-after follow-up, surveys of health professionals pre- and post- introduction of intervention, interviews with healthcare professionals	Access to assistance, Legal, Care delivery	No	Low/Medium
Kite 2016	UK, primary care, welfare rights advice in general practices	Observational, Mixed methods. Cross-sectionals data collection, client survey, interviews with clients and advisors	Access to assistance, Legal	Yes	High
Klein et al. 2013	USA, pediatric primary care centers, legal services for low income patients	Observational, Quantitative. Retrospective review of service records	Legal, Care delivery	Yes	Low/Medium
Langley et al. 2004	UK, primary care and hospital settings, welfare benefits screening and advice for adults with arthritis	Observational, Quantitative. Prospective collection of service process and outcome data	Legal	Yes	Medium/High

Levy and Payne 2006	UK, end of life care hospice, welfare advice for palliative care patients, families and carers	Observational, Quantitative. Retrospective review of service records, prospective review of referrals	Legal, Healthcare support	Yes	Low/Medium
Lishman-Peat and Brown, undated	UK, primary care, welfare benefits assessment and assistance for patients in deprived areas	Observational, Mixed methods. Retrospective review of service records, cross-sectional survey of clients including qualitative sections	Access to assistance, Legal, Prevention	Yes	Low
Mackintosh et al. 2006	UK, primary care, welfare rights assessment and assistance delivered through general practices for patients aged 60 years and above	Experimental, Quantitative. Pilot randomised controlled trial. Self-reported surveys with intervention and control groups, collected in a structured, face-to-face interview at baseline, 6, 12 and 24 months.	Legal, Health	Yes	High
Macmillan Cancer Support 2010a	UK, cancer services, welfare rights advice and assistance for people affected by cancer	Observational, Mixed methods. Unspecified	Legal, Health, Prevention, Healthcare support	No	Low
Macmillan Cancer Support 2010b	UK, cancer services, assistance with benefits and financial issues for people affected by cancer	Observational, Mixed methods. Retrospective review of service records	Legal, Health, Healthcare support	No	Low

Martin et al. 2015	USA, general hospital, lawyer embedded in inter-professional care team, for patients with high service utilization	Observational, Mixed methods. Retrospective review of service records, illustrative patient case study	Legal, Care delivery	No	Low
Mcdaid et al. 2017	UK, primary care, debt advice delivered in general practices for working aged adults	Other, Quantitative. Literature review and cost-effectiveness modelling	Healthcare support	No	Medium/High
Moffatt and Mackintosh 2009	UK, primary care, welfare rights advice delivered through general practices for people aged over 60 years who were not English	Observational, Qualitative. Cross-sectional, semi-structured interviews with clients	Access to assistance, Legal, Health, Prevention	Yes	Medium/High
Moffatt and Scambler 2008	UK, primary care, welfare rights advice in general practices for patients aged 60+	Observational, Qualitative. Prospective, Semi-structured interviews with clients pre- and post-intervention	Legal, Health, Prevention	Yes	High

Moffatt et al. 2004	UK, primary care, welfare advice delivered in general practices	Observational, Qualitative. Cross-sectional, semi-structured interviews with clients	Access to assistance, Legal, Health, Prevention, Healthcare support	Yes	High
Moffatt et al 2006	UK, primary care, welfare benefits screening and assistance delivered through general practices	Observational, Qualitative. Nested within a randomised controlled trial, semi-structured interviews with individuals in intervention and control groups	Access to assistance, Legal, Health, Prevention	Yes	High
Moffatt et al. 2010	UK, cancer services, welfare benefits assistance for cancer patients and carers	Observational, Qualitative. Cross-sectional data collection, semistructured interviews with clients (patients and carers)	Access to assistance, Legal, Health, Prevention, Care delivery	Yes	High
Moffatt et al. 2012	UK, cancer services (primary care and hospital locations), financial and welfare benefits assistance for cancer patients and carers	Observational, Mixed methods. Retrospective review of service records, semi-structured interviews	Access to assistance, Legal, Health, Prevention	Yes	High

O’Sullivan et al. 2012	USA, hospital-based asthma clinic, legal assistance to improve housing environments for asthma patients in deteriorated housing	Observational, Quantitative. Before-and-after follow-up, review of service records before and after successful intervention	Health, Healthcare support	Yes	Medium/High
O’Toole et al. 2012	USA, children's hospital clinics, legal and social welfare support for patients	Observational, Quantitative. Cross-sectional survey of medical trainees	Care delivery	Yes	Medium/High
Ollerenshaw and Camilleri 2017	Australia, community health centre, legal services for young people aged up to 25	Observational, Mixed methods. Cross-sectional surveys with clients and staff	Care delivery	Yes	Low/Medium

Palmer et al. 2010	UK, primary care, welfare advice sessions delivered in general practices	Observational, Mixed methods. Review of service records pre- and post-intervention, interviews with managers, general practitioners and advisors	Access to assistance, Health, Prevention, Healthcare support, Care delivery	No	Low/Medium
Parsonage 2013	UK, mental health inpatient units, welfare advice for people with severe mental health problems	Observational, Mixed methods. Service case study, review of service records	Legal, Prevention, Healthcare support	No	Low/Medium
Pettignano et al. 2011	USA, children's healthcare, on-site legal assistance for patients with sickle cell disease and their guardians	Observational, Quantitative. Retrospective review of service records	Legal	Yes	Medium/High
Pettignano et al. 2012	USA, children's healthcare, lawyer integrated into healthcare team, providing legal assistance for families on low incomes	Observational, Quantitative. Retrospective review of service records, cross-sectional survey of healthcare providers	Legal, Healthcare support	Yes	Low/Medium

Pettignano et al. 2013	USA, children's hospitals, on-site legal assistance for families on low incomes	Observational, Quantitative. Retrospective review of service records	Legal	Yes	Medium/High
Powell et al. 2004	UK, primary care and hospital settings, welfare benefits screening and advice for adults with arthritis	Observational, Quantitative. Prospective collection of service process and outcome data	Access to assistance, Legal, Healthcare support	Yes	Medium/High
Reading et al. 2002	UK, primary care, welfare rights advice in general practices for families with infants	Observational, Mixed methods. Before-and-after follow-up, semi-structured interviews with eligible families	Access to assistance, Legal, Health	Yes	Medium/High
Redfern Legal Centre 2015.	Australia, hospital setting	Observational, Mixed methods. Post intervention client survey, pre-and-post staff surveys	Health, Care delivery	No	Low

Regenstein et al. 2017	USA, medical-legal partnerships across the country	Observational, Quantitative. Cross-sectional survey of healthcare and legal organisations	Access to assistance, Legal, Care delivery, Healthcare support	No	Medium/High
Release 2012	UK, drug and alcohol treatment centres, legal assistance for financial and housing issues for drug users	Observational, Mixed methods. Cross-sectional survey of service users and health centre staff	Access to assistance, Legal, Health, Prevention, Care delivery, Systemic change	No	Low/Medium
Rodabaugh et al. 2010	USA, cancer institute, Legal Services Program offering legal assistance on a range of welfare and end of life issues for low income patients	Observational, Mixed methods. Retrospective review of service records, two patient case descriptions	Legal	Yes	Medium/High
Rosen Valverde et al. 2018	USA, pediatric primary care clinic, legal assistance for low income children with disabilities and their families	Observational, Quantitative. Before-and-after follow-up survey with clients	Health	Yes	Medium/High

Ryan et al. 2012	USA, family medicine clinic, legal services for low-income patients	Observational, Quantitative. Before-and-after follow-up survey with clients	Legal, Health	Yes	Medium/High
Seligman et al. 2017	UK, major trauma centre, legal services relating to personal injury for major trauma patients	Observational, Quantitative. Retrospective review of service records	Legal	Yes	Low
Sherr et al. 2002	UK, primary care, practice-based welfare rights advice sessions	Observational, Mixed methods. Cross-sectional data collection, case audits, practice manager survey, interviews with advice providers, funders perspectives	Access to assistance, Care delivery,	No	Medium/High
Sherratt et al. 2000	UK, primary care, GP practice-based welfare rights advice sessions	Observational, Mixed methods. Retrospective review of service records, interviews and focus groups	Access to assistance, Legal	Yes	Medium/High

Sinclair 2017	UK, primary care, advice on financial and housing issues for patients in deprived areas	Observational, Mixed methods. Retrospective review of service records, semi-structured interviews with staff, advisory group consultations	Access to assistance, Legal, Care delivery	No	Medium/High
Taylor et al. 2015	USA, children's hospital, legal assistance to address utility shut-offs for low income families	Observational, Quantitative. Retrospective review of service records pre- and post-intervention	Legal	Yes	Medium/High
Teufel et al. 2009	USA, independent non-profit healthcare facility, legal assistance on public benefits, personal disability, family and employment issues	Observational, Quantitative. Retrospective review of service records	Legal, Healthcare support	Yes	Low/Medium
Teufel et al. 2012	USA, rural non-profit health system, legal assistance with a variety of welfare and end of life issues for people on low incomes	Observational, Quantitative. Retrospective review of service records, comparing two time periods	Legal, Healthcare support	Yes	Medium/High

Toeg et al. 2003	UK, primary care, welfare benefits screening and advice for patients aged over 80 years	Observational, Quantitative. Prospective collection of service process and outcome data	Legal, Care delivery	Yes	Low/Medium
Tsai et al. 2017	USA, veteran's healthcare, legal advice and representation for homeless and low income veterans with mental illness	Observational, Quantitative. Retrospective review of service records, before-and-after follow-up survey of clients	Legal, Health	Yes	Medium/High
Weintraub et al. 2010	USA, children's hospital, on-site legal services for uninsured families on low incomes	Observational, Mixed methods. Before-and-after follow-up survey of clients including qualitative sections	Access to assistance, Legal, Health, Prevention, Healthcare support	Yes	Medium/High
Welsh Government 2015	UK, primary care, community care and hospital settings across Wales, co-located welfare advice sessions	Observational, Mixed methods. Retrospective review of service records, interviews with clients, consultations with project managers and directors	Access to assistance, Legal, Health, Prevention	No	Low/Medium

Woodhead et al. 2017a	UK, primary care, welfare rights advice in general practices	Observational, Qualitative. Cross-sectional data collection, semi-structured interviews with general practice staff, advisors and funders	Access to assistance, Health, Healthcare support	Yes	High
Woodhead et al. 2017b	UK, primary care, welfare advice delivered in general practices	Quasi-experimental, Quantitative. Before-and-after follow-up with comparison group via self-report survey	Access to assistance, Legal, Health, Healthcare support	Yes	High
Wright et al. 2015	UK, children's hospital, social welfare legal advice for parents and carers of children attending hospital	Observational, Mixed methods. Retrospective review of service records, post-intervention client survey, client case descriptions	Access to assistance, Legal, Health, Healthcare support, Systemic change	No	Low/Medium
Zisser and van Stone 2015	USA, health center for children with developmental disabilities, legal services assisting with access to education and resources for families and children	Observational, Mixed methods. Retrospective review of service records, cross-sectional clinician survey	Access to assistance, Legal, Systemic change, Care delivery	Yes	Low/Medium

REFERENCES

Abbott S (1999) An evaluation of the health and advice project: Its impact on the health of those using the service. Health and Community Care Research Unit.

Abbott S and Hobby L (2003) Who uses welfare benefits advice services in primary care?. Health and Social Care in the Community, 11(2):168–174. doi: 10.1046/j.1365-2524.2003.00414.x.

Abbott S, Hobby L and Cotter S (2006) What is the impact on individual health of services in general practice settings which offer welfare benefits advice?. Health and Social Care in the Community 14(1):1–8. doi: 10.1111/j.1365-2524.2005.00582.x.

Atkins D, Band M, Gardesey M, Graham L, Locke R, Mace S, Perez J, Vishnubhakta V, Waterland L (2013) Medical-Legal Partnership Pilot Project. Community Legal Aid Society, Inc. & Delaware Division of Public Health.

Bateman N (2008) Just what the doctor ordered: Welfare benefits advice and healthcare. Age Concern England.

Beck AF, Klein MD, Schaffzin JK, Tallent V, Gillam M, Kahn RS (2012) Identifying and Treating a Substandard Housing Cluster Using a Medical-Legal Partnership. Pediatrics 130:831–838. doi: 10.1542/peds.2012-0769.

Bird L (1998). Independent advice services for people with mental health problems: Needs and provision. Mental Health Care 2(4):135–139.

Burrows J, Baxter S, Baird W, Hirst J, Goyder E (2011) Citizens advice in primary care: A qualitative study of the views and experiences of service users and staff. Public Health 125:704–710. doi: 10.1016/j.puhe.2011.07.002.

Carey C, Tolopilo A (2008) Tipping the Balance: Why Legal Services are Essential to Health Care for Drug Users in Ukraine. Open Society Institute.

Carrick K, Burton K, Barclay P (2017) Forecast Social Return on Investment Analysis on the Co-location of Advice Workers with Consensual Access to Individual Medical Records in Medical Practices. Improvement Service.

Citizens Advice, Royal College of General Practitioners (2018) Advice in practice: Understanding the effects of integrating advice in primary care settings. Citizens Advice.

Citizens Advice Camden (2017) Impact and Evaluation of GP advice service in Camden.

Community Sense (2015) Advice Service for Patients of Wandle GPs Evaluation: A Final Report to Wandsworth Citizens Advice Bureaux.

Coppel DH, Packham CJ, Varnam MA (1999) Providing welfare rights advice in primary care. Public Health 113:131–135. https://doi.org/10.1016/S0033-3506(99)00137-7

Macmillan Cancer Support (2010a) Economic Impact Case Study: Durham Welfare Rights Service.

Macmillan Cancer Support (2010b) Macmillan Welfare Rights Service Durham: Economic and quality case study.

Eynon C, Dinsmore A, Dench S (2010) Lawyers in intensive care: introducing an acute legal service for patients with critical illness. Journal of The Intensive Care Society 11(2):109–111.

Fleishman SB, Retkin R, Brandfield J, Braun V (2006) The Attorney As the Newest Member of the Cancer Treatment Team. Journal of Clinical Oncolog, 24(13):2123–2126. doi: 10.1200/jco.2006.04.2788.

Focus Consultants (2017) PBLO at SickKids: A Phase II Evaluation of the Medical-Legal Partnership between Pro Bono Law Ontario and SickKids Hospital Toronto.

Frost-Gaskin M, O'Kelly R, Henderson C, Pcitti R (2003) A welfare benefits outreach project to users of community mental health services. International Journal of Social Psychiatry 49(4):251–263. doi: 10.1177/0020764003494003.

Gabbay MB, Ring A, Byng R et al. (2017) Debt counselling for depression in primary care: An adaptive randomised controlled pilot trial (DeCoDer study). Health Technology Assessment 21(35). doi: 10.3310/hta21350.

Galvin K, Sharples A, Jackson DR (2000) Citizens Advice Bureaux in general practice: an illuminative evaluation. Health and Social Care in the Community 8(4):277–282.

Greasley P, Small N (2005a) Establishing a welfare advice service in family practices: Views of advice workers and primary care staff. Family Practice 22:513–519. doi: 10.1093/fampra/cmi047.

Greasley P, Small N (2005b) Providing welfare advice in general practice: Referrals, issues and outcomes. Health and Social Care in the Community 13(3):249–258. doi: 10.1111/j.1365-2524.2005.00557.x.

Harding R, Sherr L, Singh S, Sherr A, Moorhead R (2002) Evaluation of welfare rights advice in primary care: the general practice perspective. Health and Social Care in the Community 10(6):417–422. https://doi.org/10.1046/j.1365-2524.2002.00393.x

Harding R, Sherr L, Sherr A, Moorhead R, Singh S (2003) Welfare rights advice in primary care: Prevalence processes and specialist provision. Family Practice 20(1):48–53. https://doi.org/10.1093/fampra/20.1.48

Hernández D (2016) "Extra oomph:" Addressing Housing Disparities through Medical Legal Partnership interventions. Housing Studies 31(7):871–890. doi: 10.1080/02673037.2016.1150431.

Hoskins RA, Smith LN (2002) Nurse-led welfare benefits screening in a General Practice located in a deprived area. Public Health 116: 214–220. doi: 10.1016/S0033-3506(02)90069-7.

Hoskins R, Tobin J, McMaster K, Quinn T (2005) Roll-out of a nurse-led welfare benefits screening service throughout the largest Local Health Care Co-operative in Glasgow: An evaluation study. Public Health 119: 853–861. doi: 10.1016/j.puhe.2005.03.012.

Jackson SF, Miller W, Chapman LA (2012) Hospital-Legal partnership at Toronto Hospital for Sick Children: The First Canadian Experience. Healthcare Quarterly 15(4):55–62.

Jones MK, Bloch G, Pinto AD (2017) A novel income security intervention to address poverty in a primary care setting: A retrospective chart review. BMJ Open 7. doi: 10.1136/bmjopen-2016-014270.

Justice Connect Seniors Law and cohealth (2016) Working together: Health Justice Partnership to address elder abuse. Victorian Legal Services Board & Commissioner.

Kite A (2016) Citizens Advice in GP surgeries. University of Bristol Law School.

Klein MD, Beck AF, Henize AW, Parrish DS, Fink EE, Kahn RS (2013) Doctors and Lawyers Collaborating to HeLP Children: Outcomes from a Successful Partnership between Professions. Journal of Health Care for the Poor and Underserved 24(3):1063–1073. doi: 10.1353/hpu.2013.0147.

Langley C, Memel DS, Kirwan JR, Pollock J, Hewlett S, Gubbay D, Powell J (2004) Using the health assessment questionnaire and welfare benefits advice to help people disabled through arthritis to access financial support. Rheumatology 43:863–868. doi: 10.1093/rheumatology/keh184.

Levy J, Payne M (2006) Welfare rights advocacy in a specialist health and social care setting: A service audit. British Journal of Social Work 36:323–331. doi: 10.1093/bjsw/bch366.

Lishman-Peat J, Brown G (undated) Welfare benefits take-up project in primary care in Wakefield. Benefits 10(1):45–48.

Mackintosh J, White M, Howel D, Chadwick T, Moffatt S, Deverill M, Sandell A (2006) Randomised controlled trial of welfare rights advice accessed via primary health care: Pilot study. BMC Public Health 6(162):163. doi: 10.1186/1471-2458-6-6.

Martin J, Martin A, Schultz C, Sandel M (2015) Embedding civil legal aid services in care for high-utilizing patients using medical-legal partnership. Health Affairs Blog. Doi: 10.1377/hblog20150422.047143.

Mcdaid D, Park AL, Knapp M (2017) Commissioning Cost-Effective Services for Promotion of Mental Health and Wellbeing and Prevention of Mental Ill-Health. Public Health England.

Moffatt S, Mackintosh J (2009) Older people’s experience of proactive welfare rights advice: Qualitative study of a South Asian community. Ethnicity and Health 14(1):5–25. doi: 10.1080/13557850802056455.

Moffatt S, Mackintosh J, White M, Howel D, Sandell A (2006) The acceptability and impact of a randomised controlled trial of welfare rights advice accessed via primary health care: Qualitative study. BMC Public Health 6:163. doi: 10.1186/1471-2458-6-163.

Moffatt S, Noble E, Exley C (2010) “Done more for me in a fortnight than anybody done in all me life.” How welfare rights advice can help people with cancer. BMC Health Services Research 10(1):259.

Moffatt S, Noble E, White M (2012) Addressing the financial consequences of cancer: Qualitative evaluation of a welfare rights advice service. PLoS ONE 7(8):e42979. doi: 10.1371/journal.pone.0042979.

Moffatt S, Scambler G (2008) Can welfare-rights advice targeted at older people reduce social exclusion?. Ageing and Society 28:875–899. doi: 10.1017/S0144686X08007253.

Moffatt S, White M, Stacy R, Downey D, Hudson E (2004) The impact of welfare advice in primary care: a qualitative study. Critical Public Health 14(3):295–309. doi: 10.1080/09581590400007959.

O’Sullivan MM, Brandfield J, Hoskote SS, Segal SN, Chug L, Modrykamien A, Eden E (2012) Environmental improvements brought by the legal interventions in the homes of poorly controlled inner-city adult asthmatic patients: A proof-of-concept study. Journal of Asthma 49(9):911–917. doi: 10.3109/02770903.2012.724131.

O’Toole J K, Burkhardt M C, Solan LG, Vaughn L, Klein,MD (2012) Resident confidence addressing social history: Is it influenced by availability of social and legal resources?. Clinical Pediatrics 51(7):625–631.

Ollerenshaw A, Camilleri M (2017) Health justice partnerships: Initial insights into the delivery of an integrated health and legal service for youth in regional Victoria. Rural and Remote Health 17:3975. https://doi.org/10.22605/RRH3975

Palmer S, Dalzell-Brown A, Mather K, Krska J (2010) Evaluation of the impact on GP surgeries of the Citizen’s Advice Bureau Health Outreach service. NHS Sefton.

Parsonage M (2013) Welfare advice for people who use mental health services: Developing the business case. Centre for Mental Health.

Pettignano R, Caley SB, Bliss LR (2011) Medical-Legal Partnership: Impact on Patients With Sickle Cell Disease. Pediatrics 128(6):e1482. doi: 10.1542/peds.2011-0082.

Pettignano R, Caley SB, McLaren S (2012) The Health Law Partnership: Adding a Lawyer to the Health Care Team Reduces System Costs and Improves Provider Satisfaction. Journal of Public Health Management and Practice 18(4):E1–E3. https://doi.org/10.1097/phh.0b013e31823991a9

Pettignano R, Bliss LR, Caley SB, McLaren S (2013) Can Access to a Medical-Legal Partnership Benefit Patients with Asthma who Live in an Urban Community?. Journal of Health Care for the Poor and Underserved 24:706–717. doi: 10.1353/hpu.2013.0055.

Powell JE, Langley C, Kirwan J, Gubbay D, Memel D, Pollock J, Means R, Hewlett S (2004) Welfare rights services for people disabled with arthritis integrated in primary care and hospital settings: Set-up costs and monetary benefits. Rheumatology 43:1167–1172. doi: 10.1093/rheumatology/keh278.

Reading R, Steel S, Reynolds S (2002) Citizens advice in primary care for families with young children. Child: Care, Health and Development 21(1):39–45.

doi: 10.1046/j.1365-2214.2002.00241.x.

Redfern Legal Centre (2015) Aboriginal health Justice Partnership: Evaluation of first six months of operation.

Regenstein M, Trott J, Williamson A (2017) The State of the Medical-Legal Partnership Field: Findings from the 2016 National Center for Medical-Legal Partnership Surveys. The National Center for Medical Legal Partnership.

Release (2012) Legal Outreach Surgeries Evaluation.

Rodabaugh KJ, Hammond M, Myszka D, Sandel M (2010) A Medical–Legal Partnership as a Component of a Palliative Care Model. Journal of Palliative Medicine 13(1):15–18. doi: 10.1089/jpm.2009.0203.

Rosen Valverde JN, Backstrand J, Hills L, Tanuos H (2018) Medical-Legal Partnership Impact on Parents' Perceived Stress: A Pilot Study. Behavioral Medicine. https://doi.org/10.1080/08964289.2018.1481011

Ryan AM, Kutob RM, Suther E, Hansen M, Sandel M (2012) Pilot Study of Impact of Medical-Legal Partnership Services on Patients' Perceived Stress and Wellbeing. Journal of Health Care for the Poor and Underserved 23(4):1536–1546. doi: 10.1353/hpu.2012.0179.

Seligman WH, Thompson J, Thould HE, Tan C, Dinsmore A, Lockey DJ (2017) Establishing a legal service for major trauma patients at a major trauma centre in the UK. Emergency Medicine Journal 34(9):606–607. https://doi.org/10.1136/emermed-2017-206837

Sherr L, Sherr A, Harding R, Moorhead R, Singh S (2002) A stitch in time: Accessing and funding welfare rights through Health Service Primary Care. University of London.

Sherratt M, Jones K, Middleton P (2000) A citizens' advice service in primary care: Improving patient access to benefits. Primary Health Care Research and Development 1:139–146. doi: 10.1191/146342300672823063.

Sinclair J (2017) The Deep End Advice Worker Project: embedding an advice worker in general practice settings. Glasgow Centre for Population Health.

Taylor DR, Bernstein BA, Carroll E, Oquendo E, Peyton L, Pachter LM (2015) Keeping the Heat on for Children's Health: A Successful Medical–Legal Partnership Initiative to Prevent Utility Shutoffs in Vulnerable Children. Journal of Health Care for the Poor and Underserved 26(3):676–685. doi: 10.1353/hpu.2015.0074.

Teufel JA, Brown SL, Thorne W, Goffinet DM, Clemons L (2009) Process and Impact Evaluation of a Legal Assistance and Health Care Community Partnership. Health Promotion Practice 10(3):378–385. https://doi.org/10.1177/1524839907312702

Teufel JA, Werner D, Goffinet D, Thorne W, Brown SL, Gettinger L (2012) Rural Medical-Legal Partnership and Advocacy: A Three-Year Follow-up Study. Journal of Health Care for the Poor and Underserved 23(2):705–714. doi: 10.1353/hpu.2012.0038.

Toeg D, Mercer L, Iliffe S, Lenihan P (2003) Proactive, targeted benefits advice for older people in general practice: A feasibility study. Health and Social

Care in the Community 11(2):124–128. doi: 10.1046/j.1365-2524.2003.00412.x.

Tsai J, Middleton M, Villegas J, Johnson C, Retkin R, Seidman A, Sherman S, Rosenheck RA (2017) Medical-legal partnerships at veterans affairs medical centers improved housing and psychosocial outcomes for vets. Health Affairs 36(12):2195–2203.

Weintraub D, Rodgers MA, Botcheva L, Loeb A, Knight R, Ortega K, Heymach B, Sandel M, Huffman L (2010) Pilot Study of Medical-Legal Partnership to Address Social and Legal Needs of Patients. Journal of Health Care for the Poor and Underserved 21(2):157–168.

Welsh Government (2015) An assessment of the implementation of the “Better Advice, Better Lives” scheme: Final Report.

Woodhead C, Collins H, Lomas R, Raine R (2017a) Co-located welfare advice in general practice: A realist qualitative study. Health and Social Care in the Community 25:1794–1804. doi: 10.1111/hsc.12453.

Woodhead C, Khondoker M, Lomas R, Raine R (2017b) Impact of co-located welfare advice in healthcare settings: prospective quasi-experimental controlled study. The British Journal of Psychiatry 211(6):388–395. doi: 10.1192/bjp.bp.117.202713.

Wright N, Jay M, Snell S, Holden A (2015) Great Ormond Street Hospital Citizens Advice Bureau Annual Report 2014/15. Citizens Advice Bureau.

Zisser AR, van Stone M (2015) Health, Education, Advocacy, and Law: An Innovative Approach to Improving Outcomes for Low-Income Children With Intellectual and Developmental Disabilities. Journal of Policy and Practice in Intellectual Disabilities 12(2):132–137. https://doi.org/10.1111/jppi.12115
