# Supplementary material for: International Evidence on the Impact of Health-Justice Partnerships: A Systematic Scoping Review
Source: Public Health Rev. 2021 Apr 26;42:1603976. doi: 10.3389/phrs.2021.1603976 (PMC8113986; doi:10.3389/phrs.2021.1603976)
Supplement: Supplementary file 4 [file DataSheet1.PDF]

APPENDIX 1: SEARCH STRATEGY

Table 1: Literature sources

Academic databases	Websites
Cochrane Library	Age UK
Cumulative Index to Nursing and Allied Health Literature (CINAHL)	Association of Palliative Care Social Workers
Embase	Centre for Mental Health
Health Management Information Consortium (HMIC)	Citizens Advice
Legal Journals Index	Health Justice Australia
Medline	JUSTICE
NICE Evidence	Law and Justice Foundation
PsycINFO	Law Centres Network
Social Care Institute for Excellence (SCIE)	Legal Action Group
Social Services Abstracts	Local Government Association
Social Policy and Practice	Macmillan Cancer Support
The King's Fund Library Database	Maggie’s
	Mind
	Ministry of Justice
	Money and Mental Health
	National Center for Medical-Legal Partnership
	National Institute for Health and Clinical Excellence
	NHS England
	NHS Improvement
	Open Society Foundations
	Pathway
	Public Health England
	Release
	Scottish Improvement Service
	Shelter
	Social Action for Health
	Social Care Institute for Excellence
	Social Prescribing Network
	The Health Foundation
	The King’s Fund
	The Legal Education Foundation
	Youth Access
	UCL Centre for Access to Justice

Table 2: Keyword search strategy

Concept	Search terms
**Social welfare legal advice**	(advice service* OR (legal ADJ2 advice) OR (legal ADJ2 rights) OR (legal ADJ2 service*) OR (legal ADJ2 assistance) OR (civil ADJ2 rights) OR (rights ADJ2 advice) OR (social welfare ADJ2 advice) OR (social welfare ADJ2 rights) OR social welfare OR (welfare ADJ2 advice) OR (welfare ADJ2 rights) OR (benefits ADJ2 advice) OR (debt ADJ2 advice) OR (financ* ADJ2 advice) OR (financ* ADJ2 rights) OR (housing ADJ2 advice) OR (homeless* ADJ2 advice) OR (housing ADJ2 rights) OR (employment ADJ2 advice) OR (employment ADJ2 rights) OR (education ADJ2 advice) OR (education ADJ2 rights) OR (community care ADJ2 advice) OR (community care ADJ2 rights) OR (immigration ADJ2 advice) OR (immigration ADJ2 rights) OR (family ADJ2 advice) OR (family ADJ2 rights) OR (discrimination ADJ2 advice) OR (discrimination ADJ2 rights) OR citizens advice).ti,ab,kw.
**AND**	
**Healthcare**	(health service* OR healthcare OR health-care OR health care OR medical service* OR medical care OR patient* care OR (patient* ADJ2 care) OR patient* health OR (patient* ADJ2 health) OR care delivery OR (care ADJ3 delivery) OR care pathway* OR primary care OR primary healthcare OR primary health OR general practice* OR family practice* OR secondary care OR secondary healthcare OR secondary health OR hospital* OR emergency care OR urgent care OR hospice* OR social prescri* OR health center OR health centre OR medical center OR medical centre).ti,ab,kw.
**OR**	
**Health Justice Partnership**	(medical legal partnership* OR medical-legal partnership* OR health justice partnership* OR health-justice partnership* OR hospital-legal partnership* OR health law partnership*).ti,ab,kw.

Table 3: Indexing terms

	Medical Subject Headings (MeSH)	**Other indexing terms**
**Social welfare legal advice**	• Civil rights<br>• Legal Services<br>• Social Welfare	• Community welfare services<br>• Legal processes<br>• Social welfare users<br>• Welfare rights<br>• Welfare rights advice centres<br>• Welfare services
**Healthcare**	• Delivery of health care<br>• Health Services	• Health care delivery<br>• Health care services<br>• Patient care
