# Supplementary material for: International Evidence on the Impact of Health-Justice Partnerships: A Systematic Scoping Review
Source: Public Health Rev. 2021 Apr 26;42:1603976. doi: 10.3389/phrs.2021.1603976 (PMC8113986; doi:10.3389/phrs.2021.1603976)
Supplement: Supplementary file 2 [file Table1.DOCX]

Reviewer reports:

**Reviewer #1:** This is a well written, well researched paper which should be of use to those working in the area of health justice partnerships.

Lines 200-205 have an excessive level of detail about that specific study (eg 26% of respondents reporting an outcome). Unless there is a reason for this, please edit this section to be more in line with the rest of the paper.

- *Response: Thank you for the comment, I have reduced the amount of detail in this section as suggested.*

**Reviewer #2:** This scoping review is a timely and important contribution to the literature on Health Justice Partnerships. While evidence reviews have been done previously at the country level (e.g. the U.S.), this is the first synthesis of international evidence. As HJPs continue to expand across the globe, understanding the evidence and gaps in knowledge is critical. The paper is well written and organized and clearly defines and articulates its methods and limitations.

My main suggestions focus on providing slightly more discussion about contextual differences across countries:

- As a reader, I was curious about which country the evidence discussed was generated from. The authors note quite frequently when a study was from the UK, but not as often when it was from a different country. While it may be cumbersome to indicate in every instance where a study was conducted, in some instances, additional context would be helpful. For example, it would have been helpful to the reader to know that the studies in lines 208-214 were all from the U.S.
  - *Response: Thank you for highlighting this, I have added the countries in the paragraph you mention and also checked the rest of the text for places this could be specified more clearly. In some sentences the findings of studies are grouped, so I have added ‘international’ to make it clear this includes a range of countries.*
- The discussion of "support for health care services" on page 10 needs additional context regarding the goals of the different studies. In some cases providing legal services promotes access to needed health care, particularly preventive primary care services. In other cases, legal services may help to reduce utilization of health care services, such as hospitalization and emergency room services by preventing more serious health problems. More explicit discussion of this distinction would be helpful when describing the studies. Particularly in the U.S., studies have focused on the ways in which HJPs can support early prevention, reducing more expensive and sometimes unnecessary health care utilization.
  - *Response: Thank you for the suggestion, this point certainly needs bringing out. I have added extra detail in that section, including a separate paragraph with extra references (lines 263-270). I have also added a comment in the discussion to address this (lines 341-343).*
- Similarly, the authors note that in U.S. system where there is not universal access to health insurance, some studies of HJPs have focused on how acquiring insurance for patients can support return on investment for their health care partners. This context could be made more explicit since acquiring insurance for patients in the U.S. has multiple goals - promoting access to health care, especially primary care, reducing more expensive and less preventive care such as emergency room services, and reducing unreimbursed care for health care partners. This is distinct from other countries.
  - *Response: Thank you, I have addressed this alongside the point above, both in the results and discussion sections.*
- Regarding the discussion of studies addressing inequalities. It may be worthwhile to reiterate for the reader that while few studies have explicitly addressed reduction in health inequities, the populations targeted by HJPs are those with low socioeconomic status; therefore, the benefits from HJPs most often accrue to those with low SES. Of course, population level studies are needed to assess the direct effect of HJPs on health and social inequality.
  - *Response: Thank you, this is an important point which I have added to the discussion as suggested (please see lines 335-337)*
- On page 15, lines 319-324, the authors should include discussion of an additional study from the U.S. that is not included in their review:

Robert Sege, Genevieve Preer, Samantha J. Morton, Howard Cabral, Oluwatomisin Morakinyo, Vonne Lee, Catarina Abreu, Edward De Vos and Margot Kaplan-Sanoff. Medical-Legal Strategies to Improve Infant Health Care: A Randomized Trial. Pediatrics July 2015, 136 (1) 97-106; DOI: <https://eur01.safelinks.protection.outlook.com/?url=https%3A%2F%2Fdoi.org%2F10.1542%2Fpeds.2014-2955&amp;data=04%7C01%7C%7C7b87068d4d6a4e5018a008d883424aaf%7C1faf88fea9984c5b93c9210a11d9a5c2%7C0%7C1%7C637403668041304004%7CUnknown%7CTWFpbGZsb3d8eyJWIjoiMC4wLjAwMDAiLCJQIjoiV2luMzIiLCJBTiI6Ik1haWwiLCJXVCI6Mn0%3D%7C2000&amp;sdata=oD3npWclc8URiwqZcXlj8BnhuiI7T4Fc8U08uNSba%2Fk%3D&amp;reserved=0>. <https://eur01.safelinks.protection.outlook.com/?url=https%3A%2F%2Fpediatrics.aappublications.org%2Fcontent%2F136%2F1%2F97.long&amp;data=04%7C01%7C%7C7b87068d4d6a4e5018a008d883424aaf%7C1faf88fea9984c5b93c9210a11d9a5c2%7C0%7C1%7C637403668041314007%7CUnknown%7CTWFpbGZsb3d8eyJWIjoiMC4wLjAwMDAiLCJQIjoiV2luMzIiLCJBTiI6Ik1haWwiLCJXVCI6Mn0%3D%7C2000&amp;sdata=3covnzncwUym%2BVK4%2Ft1d2PwrVnYBlGyHl78JyRUsb7I%3D&amp;reserved=0>

- *Response: Thank you for highlighting this study. It was considered during the screening process but not included for the following reason: the study took place in a system that included Medical-Legal Partnership services, but examined the impact of the family support worker role rather than the MLP. The impacts observed could not be attributed to legal assistance directly, as the intervention was multi-faceted and the control group also had access to MLP services.*
- Finally, the authors may wish to include a few sentences in their conclusion regarding how this scoping review can inform where there are gaps in knowledge and potential future research goals.
  - *Response: Thank you, this is a helpful suggestion and I have added an additional paragraph in the conclusion to address this.*
